# Targeting LSD1 suppresses stem cell-like properties and sensitizes head and neck squamous cell carcinoma to PD-1 blockade

**DOI:** 10.1038/s41419-021-04297-0

**Published:** 2021-10-23

**Authors:** Yong Han, Shengming Xu, Weimin Ye, Yang Wang, Xiangkai Zhang, Jiong Deng, Zhiyuan Zhang, Liu Liu, Shuli Liu

**Affiliations:** 1 Department of Oral and Maxillofacial-Head and Neck Oncology, Shanghai Ninth People’s Hospital, College of Stomatology, Shanghai Jiao Tong University School of Medicine, Shanghai, China; 2 National Clinical Research Center for Oral Diseases, Shanghai, China; 3 Shanghai Key Laboratory of Stomatology & Shanghai Research Institute of Stomatology, Shanghai, China; 4 Research Unit of Oral and Maxillofacial Regenerative Medicine, Chinese Academy of Medical Sciences, Shanghai, China; 5 Key Laboratory of Cell Differentiation and Apoptosis of Chinese Minister of Education, Shanghai Jiao Tong University School of Medicine, Shanghai, China

**Keywords:** Oral cancer, Stem-cell research

## Abstract

Head and neck squamous cell carcinoma (HNSCC) is a highly aggressive tumor with poor clinical outcomes due to recurrence, metastasis, and treatment resistance. Cancer stem cells (CSCs), a small population among tumor cells, are proposed to be responsible for tumor initiation, progression, metastasis, drug resistance, and recurrence. Here we show that high LSD1 expression was a predictor of poor prognosis for HNSCC patients. We found that high expression of LSD1 is essential for the maintenance of the CSC properties by regulating Bmi-1 expression. Moreover, tumor LSD1 ablation suppresses CSC-like characteristics in vitro and inhibits tumorigenicity in vivo in immune-deficient xenografts. However, this deletion induces the upregulation of PDL1 levels, which compromises antitumor immunity and reduces antitumor efficacy in an immune-competent mouse model. Functionally, the combination of LSD1 inhibitor and anti-PD-1 monoclonal antibody can overcome tumor immune evasion and greatly inhibit tumor growth, which was associated with reduced Ki-67 level and augmented CD8^+^ T cell infiltration in immunocompetent tumor-bearing mouse models. In summary, these findings provide a novel and promising combined strategy for the treatment of HNSCC using a combination of LSD1 inhibition and PD-1 blockade.

## Introduction

Head and neck squamous cell carcinoma (HNSCC) is an aggressive malignancy with a low 5-year survival rate and poor prognosis [1]. Despite a variety of advances in combined modality treatments over the past three decades, the 5-year survival rate for HNSCC patients remains unsatisfactory, largely due to uncontrolled local recurrence and metastasis [2]. Cancer stem cells (CSCs), also known as cancer-initiating cells, have been proposed to play vital roles in treatment resistance and metastasis [3]. Moreover, given their tumor-initiating capacity, surviving CSCs are qualified to serve as precursors of new tumor masses, ultimately leading to local recurrence [4]. Thus, it may be necessary to target and eliminate these cells to eradicate cancers. However, the acceptance of the CSC concept has caused some to argue that the usually limited efficacy of conventional anticancer therapies is attributable to these therapies targeting the bulk population of non-CSCs within individual tumors but not eliminating the rare subpopulation of CSCs [5]. Thus, highlighting the role of CSCs and identifying new therapeutic targeting options for eliminating CSCs are important for the treatment of HNSCC.

Immune checkpoint blockade has been actively studied in recent years, and immune checkpoint inhibitors have produced tremendous clinical benefits in several malignancies, such as recurrent/metastatic HNSCC [6]. However, a majority of cancer patients do not respond to anti-programmed cell death protein (ligand) 1 (anti-PD(L)-1) therapy due to multiple immunosuppressive mechanisms in the tumor microenvironment (TME), including dysfunctional T cells and a lack of T cell infiltration or tumor recognition by T cells [7]. CSCs are more resistant to immunological control than non-CSCs, and cancer immunosurveillance enriches a subpopulation of cancer cells with stem-like properties. Recent studies further suggest that enriched PDL1 in CSCs may contribute to CSC immune evasion [8]. These findings suggest potential links between CSCs and antitumor immunity. Very recently, it has been shown that CSCs directly inhibit cytotoxic T cell activity and mediate tumor resistance to adoptive cytotoxic T cell transfer-based immunotherapy by expressing CD80 [9]. These studies suggest that targeting CSCs may be critical for improving the efficacy of immunotherapy and preventing tumor relapse.

Lysine-specific demethylase 1 (LSD1, also known as KDM1A) is a key component of various transcriptional corepressor complexes that selectively removes the methyl group from H3K4me1/2 and thus mediates gene repression [10]. It has also been identified as a bona fide oncogene overexpressed across a broad spectrum of malignancies and as a druggable target with translational potential. Genetic depletion or pharmacological inhibition of LSD1 potently inhibits cell proliferation, cell migration, epithelial-to-mesenchymal transition (EMT), and chemoresistance, ultimately restraining cancer growth and metastasis [11, 12]. Interestingly, recent studies have implicated LSD1 in the regulation of the pool of CSCs in different leukemias and solid tumors [13]. More surprisingly, recent studies indicate that LSD1 ablation can trigger antitumor immunity through induced expression of endogenous retroviruses and reduced expression of the RNA-induced silencing complex [14]. These studies also show that LSD1 inhibition overcomes resistance to checkpoint blockade therapy in vivo by increasing tumor immunogenicity and T cell infiltration [14, 15]. However, the precise mechanisms by which LSD1 mediates its effects on HNSCC cancer stemness and how the full spectrum of chromatin regulators modulates tumor responses to cancer immunotherapy are largely unknown. Here we investigated the crosstalk between LSD1 inhibition and immune checkpoints, in particular, the PDL1/PD-1 axis, and further explored a new mechanism-driven clinical trial for PD-1 blockade-based combination therapy with LSD1 inhibitors.

## Materials and methods

### Cell cultures, plasmids, and reagents

The HNSCC-derived cancer cell lines HN30, HN4, HN6, HN12, and HN13 were kindly provided by the University of Maryland, School of Dentistry. The HEK293 and CAL27 cell lines were purchased from the American Type Culture Collection (ATCC, Manassas, VA, USA). The human immortalized oral epithelial cell (HIOEC) line was established by our laboratory previously. HN4, HN6, HN12, HN13, HN30, HEK293, and CAL27 cells were cultured in Dulbecco’s modified Eagle’s medium (Gibco) supplemented with 10% fetal bovine serum, 1% glutamine, and 1% penicillin–streptomycin. HIOECs were cultured in defined keratinocyte serum-free medium. All cells were maintained in a humidified atmosphere of 5% CO_2_ at 37 °C.

LSD1 and Bmi-1 plasmids were kindly provided by Dr. Binhua P. Zhou (University of Kentucky). LSD1-specific short hairpin RNA (shRNA) expression plasmids were purchased from Shanghai Era Biotech (Shanghai, China). Deletion mutants of LSD1 and Bmi-1 plasmids were constructed as described previously [16, 17]. All sequences were verified by DNA sequencing. TCP was purchased from Sigma-Aldrich (St. Louis, MO, USA). ORY-1001 and SP2509 were purchased from Selleckchem (Houston, USA). An anti-GAPDH antibody was obtained from Santa Cruz Biotechnology (Santa Cruz, CA). Antibodies against LSD1, CD44, Bmi-1, Oct4, Sox2, HA, Flag, PDL1, and H3K4me2 were purchased from Cell Signaling Technology (Danvers, MA, USA).

### Western blotting and immunoprecipitation (IP)

The experimental protocols for western blotting and IP were performed as described previously [18]. Briefly, RIPA lysis buffer was used to lyse cells on ice. IP was performed with 2 µg of antibodies against HA, Flag, LSD1, or Bmi-1 or normal IgG (as a negative control) in 1.0 mg of whole-cell lysates. Then cell lysates or immunoprecipitated cellular proteins were separated by sodium dodecyl sulfate-polyacrylamide gel electrophoresis in an acrylamide gel and transferred to a nitrocellulose membrane for further immunoblotting.

### Tumorsphere culture

The experimental protocol for tumorsphere formation was described previously [19]. Single-cell suspensions were prepared at a density of 4000 cells per milliliter and seeded into 6-well plates (2.0 mL per plate) coated with 1.2% poly-Hema (Sigma-Aldrich). Suspension culture was continued for 1–2 weeks until tumorspheres formed. Tumorspheres with a diameter >50 μM were counted. Recorded data represent the colony number obtained from ten separate views using a microscope. Experiments were repeated three times with duplicates included in each experiment.

### Immunofluorescence (IF)

The IF protocol was performed as described previously [20]. Cultured cancer cells were rinsed with phosphate-buffered saline (PBS) three times and fixed with 3.7% formaldehyde. Then the cells were permeabilized with 0.1% Triton X-100. After 1 h of blocking with 1% bovine serum albumin, the cells were then incubated with a primary antibody overnight. Then the cells were washed and incubated (in the dark) with an Alexa Fluor 488- or Alexa Fluor 594-conjugated donkey anti-rabbit IgG antibody (Invitrogen, Grand Island, NY) for 1 h at room temperature. The cells were washed with PBS (containing 0.02% Tween 20), stained by mounting on a slide with aqueous mounting medium containing 0.5 mg/mL 4′,6-diamidino-2-phenylindole and examined with a fluorescence microscope.

### Primary HNSCC samples

Formalin-fixed, paraffin-embedded material was obtained from surgically resected HNSCC specimens at the Ninth People’s Hospital (Shanghai, China). In total, 85 primary HNSCC patients who had not undergone prior radiotherapy or chemotherapy treatment were enrolled in this study. The average age of the patients was 57.01 years, ranging from 18 to 83 years. For each neoplastic tissue sample, histopathological diagnosis was performed according to the criteria of the World Health Organization. The Tumor, Node, Metastasis classification of the International Union against Cancer was used to determine the clinicopathological staging. Our study was approved by the Ethics Committee of the Shanghai Ninth People’s Hospital, Shanghai Jiao Tong University School of Medicine (Shanghai, China). We also carried out our study according to the recommendations of the Declaration of Helsinki.

### Immunohistochemical staining and evaluation

Four-micron tissue sections were deparaffinized, rehydrated, and washed with PBS for immunohistochemical analysis. After antigens were retrieved by adding 0.01 mol/L citrate buffer (pH 6; Dako Cytomation), the slides were then incubated in a steamer for 30 min. The endogenous activity of each sample was blocked in a 3% hydrogen peroxide/PBS and avidin/biotin solution (Zymed Laboratories), and then the samples were incubated with anti-human LSD1 and Bmi-1 antibodies (Cell Signaling Technology) overnight at 4 °C. The slides were then rinsed and incubated with a biotinylated antibody and labeled with streptavidin–peroxidase from labeled streptavidin–biotin kits (Dako Cytomation). The samples were then stained with a 3,3′-diaminobenzidine tetrahydrochloride solution and counterstained with Mayer’s hematoxylin for 2 min. The LSD1 and Bmi-1 staining indexes were calculated as the product of the staining intensity and percentage. Images were examined by two independent observers blinded to the experiment.

### Aldefluor assay

We used an Aldefluor Kit (Stem Cell Technologies, Durham) to isolate the population with high aldehyde dehydrogenase (ALDH) activity in accordance with the manufacturer’s guidance. HNSCC cells were suspended in ALDH substrate buffer and incubated in a 37 °C water bath for 50 min. Cells from each sample treated with 50 mmol/L diethylaminobenzaldehyde were used as negative controls. Following the incubation, all samples were centrifuged for 5 min at 250 × *g*, and the supernatant was removed. The cell pellets were resuspended in 0.5 mL of Aldefluor assay buffer and stored on ice. Then flow cytometry was performed to evaluate all samples.

### Flow cytometric analysis

Fresh tumors were obtained to examine immune cell populations. Tumors were mechanically dissociated using a gentle MACSTM dissociator (Miltenyi Biotec). RBC lysis buffer (BD Biosciences) was applied to isolate lymphocytes from the peripheral blood. Gradient separation was performed using a Percoll solution (GE Healthcare Life Sciences) to isolate lymphocytes from tumors. Freshly isolated cells were preincubated with anti-mouse CD16/CD32 monoclonal antibodies (mAbs; 2.4G2, BD Biosciences) to block the binding of antibodies to FcγIII/II receptor and then stained with a fixable viability dye (Thermo Fisher Scientific) prior to antibody staining to exclude dead cells. The cells were then stained with fluorochrome-conjugated antibodies for 15 min at room temperature. For intracellular staining, cells were incubated with a fixation/permeabilization solution (Thermo Fisher Scientific) and stained with fluorochrome-conjugated antibodies. For intracellular cytokine staining, cells were stimulated in the presence of brefeldin A (GolgiPlug, BD Biosciences) and monensin (GolgiSTOP, BD Biosciences) for 6 h according to the manufacturer’s instructions. The antibodies for fluorescence-activated cell sorting (FACS) analysis were obtained from BD Biosciences (CD3, 145-2C11; PD-1, J43; IFN-γ, XMG1.2) and BioLegend (CD45, 30-F11; CD8, 53-6.7; CD4, GK1.5; Foxp3, MF14; CD25, PC61; Gr1, RB6-8C5; CD11b, M1/70; CD11c, N418). All flow cytometric analyses were performed using an LSR II system (BD Biosciences) and FlowJo software (Tree Star Inc.).

### Detection of cell-surface PDL1

For detection of cell-surface PDL1, tumor cells were suspended in 100 µL of cell staining buffer (#420201, BioLegend) and incubated with an allophycocyanin-conjugated anti-mouse PDL1 antibody (MIH5, BD Biosciences) at room temperature for 30 min. After washing in staining buffer, the stained cells were analyzed by FACS (BD Biosciences).

### Terminal deoxynucleotidyl transferase-mediated dUTP-fluorescein nick end labeling (TUNEL)

Paraffin-embedded tumor sections from SCC7 inoculated mice were analyzed by TUNEL assay, using the one-step TUNEL Kit (Beyotime Biotech, Jiangsu, China). Cells were observed under a fluorescence microscope and the cells with green fluorescence were defined as apoptotic cells.

### Animal studies

Animal experiments were approved by the Animal Ethics Committee of the Ninth People’s Hospital. All animal procedures were performed according to guidelines approved by the Shanghai Jiao Tong University School of Medicine. For the in vivo study, female specific pathogen-free (SPF) BALB/c nude mice (4 weeks old) were purchased from the Shanghai Laboratory Animal Center (Shanghai, China). All procedures were approved by the Laboratory Animal Care and Use Committees of our hospital. A tumor xenograft model was established with HN12 and HN30 cells, which exhibit high LSD1 expression in vitro. The cells (5 × 10^6^ cells/100 μL PBS) were subcutaneously inoculated into the flanks of nude mice, and tumor sizes were monitored three times per week.

For the immunocompetent mouse study, female C3H/He mice aged 6–8 weeks were purchased from Vital River Laboratory Technology (Beijing). All mice were maintained under SPF conditions in the animal facilities of the Ninth People’s Hospital. SCC7 cells (5 × 10^4^) were subcutaneously injected into the right flank. When tumors became palpable, the tumor-bearing mice were treated intraperitoneally with an anti-PD-1 antibody (200 µg/mouse, BioXCell) every 3 days as reported before [21]. SP2509 was administered by daily oral gavage at a dose of 50 mg/kg [22]. Tumor volumes were determined by measuring the length (*a*), width (*b*), and height (*h*) and calculating the tumor volume as *abh*/2.

### Statistics

Experiments were repeated at least two times. Results are expressed as mean ± SD or SEM as indicated. A two-tailed Student’s *t* test was used for intergroup comparisons. *P* value < 0.05 was considered statistically significant.

## Results

### LSD1 is aberrantly expressed in HNSCC and associated with a poor prognosis

**Fig. 1:**
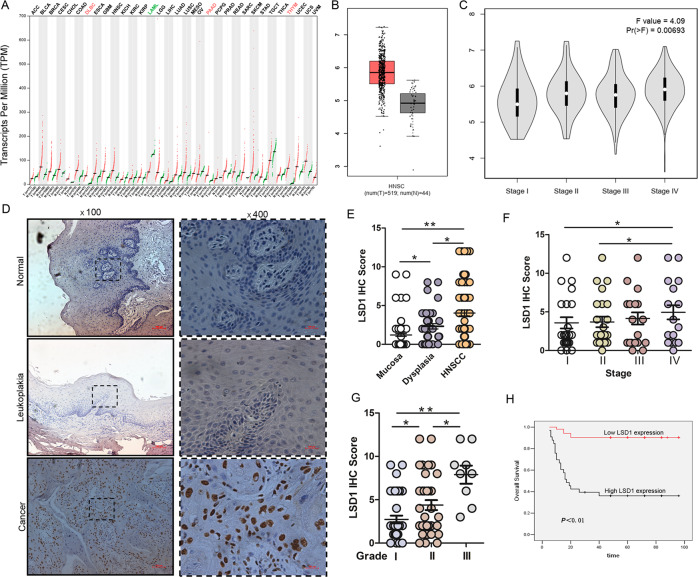
High LSD1 expression predicts a poor prognosis in HNSCC patients. **A** The expression levels of LSD1 were detected in different types of cancers from the GEPIA public database. **B** The GEPIA database revealed that LSD1 expression was significantly upregulated in HNSCC patients. **C** Correlation between the expression level of LSD1 and the pathological stage of HNSCC patients (GEPIA). **D** Representative images of LSD1 expression in normal tissues, leukoplakia tissues, and HNSCC tissues via immunohistochemical (IHC) staining. Scale bar: 100 μm. **E** IHC scores for LSD1 expression in normal, leukoplakia, and HNSCC tissues. **F** IHC scores for LSD1 expression in HNSCC samples in different TNM stages. **G** IHC scores for LSD1 expression in HNSCC with different grades. **H** High LSD1 expression significantly correlates with poor survival in HNSCC patients. The survival rates of patients with LSD1-low and LSD1-high tumors (*P* < 0.01) were determined using Kaplan–Meier survival test. (*p < 0.05, **p < 0.01).

To explore the role of LSD1 in HNSCC progression, we first compared the mRNA expression of LSD1 between tumor and normal tissues by using a GEPIA (Gene Expression Profiling Interactive Analysis) dataset [23]. We found that LSD1 is highly expressed in the majority of human cancers (Fig. 1A), including HNSCC. The results indicated that the expression level of LSD1 was higher in HNSCC cancer tissues than in normal tissues (Fig. 1B). Then we analyzed the correlation between the expression level of LSD1 and tumor stage in HNSCC. LSD1 expression was positively correlated with HNSCC stage (*P* < 0.001; Fig. 1C). To further explore the role of LSD1 in HNSCC progression, we next used immunohistochemistry to detect the expression level of LSD1 in 85 cases of primary HNSCC tissues, 30 cases of normal oral mucosal tissues, and 40 cases of leukoplakia (precancerous lesion) patient tissues. The HNSCC patient demographics and clinicopathological data are shown in Table 1. We found that the LSD1 expression level in HNSCC tissues was significantly higher than that in normal oral tissues and leukoplakia tissues and that there were significant differences between normal and leukoplakia tissues and between leukoplakia and HNSCC tissues (Fig. 1D, E). We next examined the relationships of LSD1 expression with clinicopathological parameters and patient survival. We found that HNSCC patients with an advanced stage and a high pathological grade (II and III) displayed higher LSD1 expression levels than those with an early stage and pathological grade (Fig. 1F, G). Next, we examined the prognostic value of LSD1 expression in HNSCC; surprisingly, Kaplan–Meier analysis showed that patients with higher LSD1 expression levels had poorer outcomes, and vice versa (Fig. 1H). Altogether, these data indicate that LSD1 overexpression is associated with a poor prognosis in HNSCC patients.

**Table 1:** Baseline characteristics of HNSCC patients included in the study.

Characteristics	Patients	
	No.	Percent (%)
Age, years		
≤60	53	62.4
>60	32	37.6
Sex		
Male	40	47.1
Female	45	52.9
T—primary tumor size		
T1	24	28.2
T2	35	41.2
T3	14	16.5
T4	12	14.1
N—regional lymph node		
Negative	55	64.7
Positive	30	35.3
TNM stage		
I	24	28.2
II	23	27.1
III	20	23.5
IV	18	21.2
Histopathological type		
Grade 1	37	43.5
Grade 2	39	45.9
Grade 3	9	10.6
Smoking history		
Yes	29	34.1
No	56	65.9
Alcohol history		
Yes	29	34.1
No	56	65.9
Total	85	100

### Elevated LSD1 is important for the maintenance of CSC phenotypes in HNSCC

**Fig. 2:**
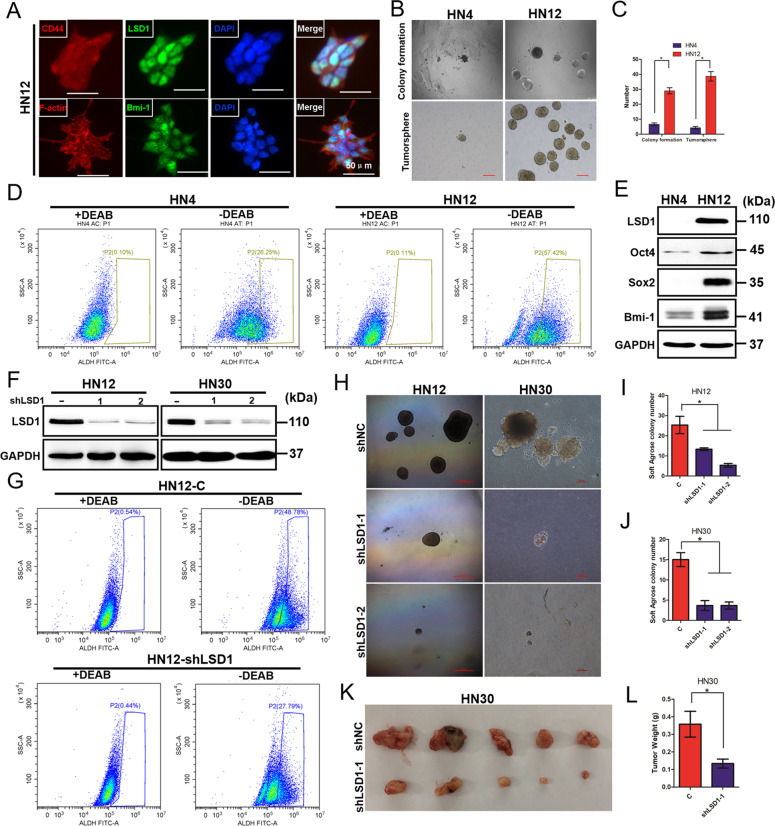
Elevated LSD1 is important for the maintenance of CSC phenotypes in HNSCC. **A** Representative coimmunofluorescence staining images for LSD1 and the CSC markers Bmi-1 and CD44 in HN12 cells (scale bar, 50 μm). **B** Representative images of colony formation and tumorsphere formation by HN4 and HN12 cells. Scale bar = 200 μm. **C** Measurement of colony formation and tumorsphere formation (mean ± SD from three separate experiments) by HN12 and HN4 cells. **D** An Aldefluor assay was conducted with HN4 and HN12 cells, and the percentage of ALDH-high cells was quantified by flow cytometry. **E** The expression of LSD1 and CSC-related proteins in HN4 and HN12 cells was detected by western blotting. **F** Western blot analysis of LSD1 in HN12 and HN30 cells stably expressing a control vector or LSD1-specific shRNA. **G** An ALDEFLUOR assay was conducted with HN12 LSD1-knockdown and control cells, and the percentage of ALDH-high cells was quantified by flow cytometry. **H** Images of tumorsphere formation by HN12 and HN30 cells stably expressing a control vector or LSD1-specific shRNA. Scale bar = 200 μm. **I** The graph demonstrates the mean ± SD for the tumorsphere number of HN12 cells stably expressing a control vector or LSD1-specific shRNA from three separate experiments. **J** The graph demonstrates the mean ± SD for the tumorsphere number of HN30 cells stably expressing a control vector or LSD1-specific shRNA from three separate experiments. **K**, **L** HN30 cells stably transfected with control or LSD1-specific shRNAs were injected into nude mice. Tumor growth was monitored every 3 days; tumor size and weight were recorded. The data are presented as mean ± SEM from five mice. *P < 0.05 vector control cells compared with corresponding LSD1-knockdown cells.

Accumulating evidence has revealed that epigenetic modulating agents play dramatic roles in cellular reprogramming, in particular reversing cancer stemness characteristics, such as self-renewal and chemoresistance. It has been reported that LSD1 plays a well-established role in maintaining the self-renewal of normal hematopoietic and neuronal stem cells [13]. Thus, we inferred that LSD1 may be related to CSC properties in HNSCC. As our previous study reported, the HN4 and HN12 cell lines were derived from the same patient, with the HN12 cell line being a nodal metastatic subclone of HN4 cells. HN12 cells exhibit more CSC characteristics, such as an increased ability to form tumorspheres and the expression of stem cell-like markers [19]. To determine whether LSD1 enhances CSC characteristics in HNSCC, we performed a series of experiments with the HN4 and HN12 cell lines. We found that HN12 cells exhibited high coimmunofluorescence staining for LSD1 and several well-known CSC markers (Fig. 2A), which correlated with higher tumorsphere and colony-formation abilities (Fig. 2B, C), suggesting that LSD1 is highly expressed in cells with strong stemness properties. This elevated expression of LSD1 in HN12 cells was validated by an ALDH enzymatic activity assay and western blotting (Fig. 2D, E). To further determine whether LSD1 is essential for the maintenance of CSC-like characteristics, we examined and compared the biochemical and biological characteristics of stable shRNA-LSD1 transfectants (knockdown of LSD1 expression), HN12 and HN30 cells. As expected, knockdown of LSD1 expression significantly suppressed ALDH activity compared to control expression (Fig. 2F, G). Moreover, the transfected cells showed suppressed colony formation compared with control HN12 and HN30 cells (Fig. 2H–J). To further extend our findings in vivo, we analyzed whether the knockdown of LSD1 affects tumorigenicity by using a xenograft mouse model. Strikingly, knockdown of LSD1 significantly inhibited HN30 tumor growth in vivo (Fig. 2K, L). Taken together, these observations demonstrate that LSD1 is essential for the maintenance of CSC-like characteristics in HNSCC.

### Bmi-1 is required for LSD1-driven HNSCC oncogenesis

**Fig. 3:**
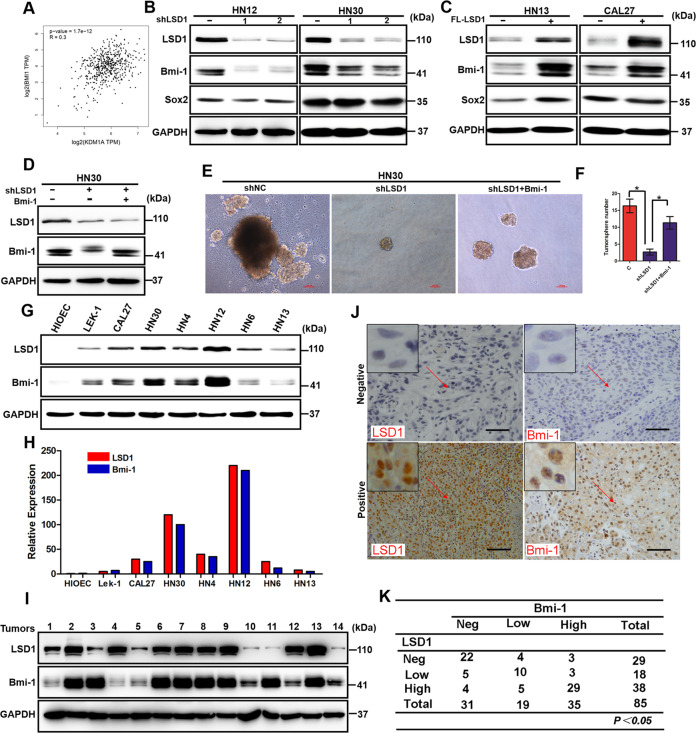
Bmi-1 is required for LSD1-driven HNSCC oncogenesis. **A** The correlations between the expression levels of LSD1 and Bmi-1 in HNSCC. **B** Western blot analysis of LSD1, Bmi-1, and Sox2 expression in the control and LSD1-silenced groups of HN12 and HN30 cells. **C** Western blot analysis of LSD1, Bmi-1, and Sox2 expression in the control and LSD1-overexpressing groups of HN13 and CAL27 cells. **D** Western blot analysis of LSD1 and Bmi-1 expression in the control, LSD1-silenced, and Bmi-1-rescued groups of HN30 cells. **E**, **F** Representative images and quantitative analysis of a sphere-formation assay performed with the control, LSD1-silenced, and Bmi-1 rescued groups of HN30 cells. *P < 0.01, error bar values represent the SD. **G** The expression of endogenous LSD1 and Bmi-1 in various tumor cell lines was analyzed by western blotting. **H** Values are normalized to the value of GAPDH. **I** The expression of LSD1 and Bmi-1 in 14 fresh-frozen human HNSCC tumors was examined by western blotting. **J**, **K** Eighty-five HNSCC specimens were immunostained using antibodies against LSD1 and Bmi-1 and control serum (data not shown). Representative images of immunohistochemical (IHC) staining of the same tumor samples are shown in **J**, and the statistical analysis is shown in **K**.

The abovementioned results prompted us to further explore the mechanism involving LSD1 in the maintenance of CSC-like characteristics in HNSCC. As reported, CSCs in HNSCC are characterized by the expression of CD44, CD133, ALDH1A1, CD24, Bmi-1, Sox2, Oct4, C-Met, NANOG, KLF4, and Lin28 [24]. Thus, we first analyzed the expression correlations of these genes with LSD1 by using GEPIA. Surprisingly, Bmi-1 exhibited the highest positive correlation with LSD1 (Fig. 3A), and we observed a positive correlation between LSD1 and Bmi-1 by Spearman correlation analysis. However, the other genes showed no significant correlations with LSD1 (Fig. S1). Furthermore, the Bmi-1 protein level was reduced when LSD1 was knocked down in both HN12 and HN30 cells (Fig. 3B), whereas it was increased in both HN13 and CAL27 cells when LSD1 was stably transfected (Fig. 3C). To further examine the role of Bmi-1 in LSD1-driven oncogenesis, we first knocked down LSD1 and then overexpressed Bmi-1 in HN30 cells (Fig. 3D). We then performed tumorsphere-formation assays and found that LSD1 knockdown significantly reduced the number and size of tumorspheres made of HN30 cells, as expected. However, Bmi-1 overexpression dramatically enhanced both the number and size of tumorspheres again (Fig. 3E, F). To determine whether the expression of LSD1 and Bmi-1 is correlated in HNSCC, we examined their expression in multiple HNSCC cell lines and tumor specimens. As expected, LSD1 and Bmi-1 levels were coordinately high in HNSCC cell lines (Fig. 3G, H). Furthermore, we examined the protein levels of LSD1 and Bmi-1 in 14 fresh-frozen HNSCC tumor samples. Although two samples did not match well (samples 4 and 11, Fig. 3I), the majority of the tumor samples showed a positive correlation for the expression of LSD1 and Bmi-1 at the protein level. The correlation of LSD1 and Bmi-1 was further validated by examining the expression of these two molecules in 85 primary HNSCC tumor samples using immunohistochemical staining. We found a significant correlation between LSD1 and Bmi-1 in terms of protein intensity and distribution in the HNSCC tumor specimens (Fig. 3J, K). These data support our findings in cell culture experiments and further strengthen our conclusion that Bmi-1 is critical for LSD1-driven HNSCC oncogenesis.

### Bmi-1 interacts with LSD1

**Fig. 4:**
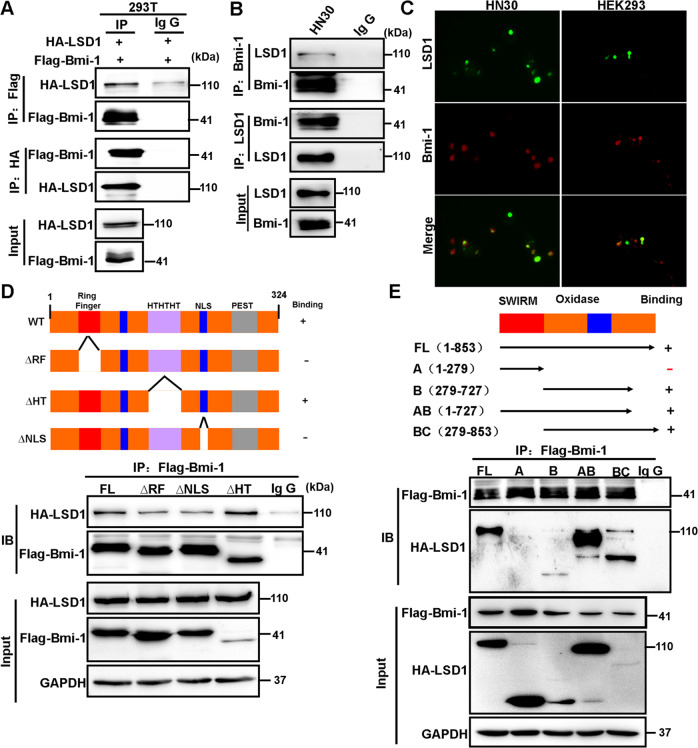
LSD1 interacts with Bmi-1. **A** HA-LSD1 was coexpressed with Flag-Bmi-1 in HEK293 cells. LSD1 and Bmi-1 were immunoprecipitated with an anti-HA or anti-Flag antibody, respectively, and the associated Bmi-1 and LSD1 were analyzed by western blotting using either an anti-HA or an anti-Flag antibody. **B** Endogenous LSD1 and Bmi-1 were immunoprecipitated from HN30 cells, and bound endogenous Bmi-1 and LSD1 were examined by western blotting. **C** The cellular location of LSD1 (green) and Bmi-1 (red) was examined by immunofluorescence staining of HN30 and HEK293 cells. **D** Schematic diagram showing the structure of Bmi-1 and deletion constructs used (top panel). Flag-tagged full-length or deletion mutants of Bmi-1 were coexpressed with HA-LSD1 in HEK293 cells. Extracts were immunoprecipitated with an anti-Flag antibody, and bound LSD1 was examined by western blotting using an anti-HA antibody. **E** Schematic diagram showing the structure of LSD1 and deletion constructs used (top panel). HA-tagged full-length or deletion mutants of LSD1 were coexpressed with Flag-Bmi-1 in HEK293 cells. Extracts were immunoprecipitated with an anti-Flag antibody, and bound LSD1 was examined by western blotting using an anti-HA antibody.

To investigate whether LSD1 interacts with Bmi-1, we coexpressed HA-LSD1 and Flag-Bmi-1 in HEK293 cells. After immunoprecipitating Bmi-1, we detected the associated LSD1, and vice versa (Fig. 4A). We also immunoprecipitated endogenous LSD1 or Bmi-1 from HN30 cells and detected the presence of endogenous Bmi-1 or LSD1, respectively (Fig. 4B). The LSD1/Bmi-1 interaction was further supported by IF analysis, as LSD1 colocalized with Bmi-1 in the nucleus of HN30 and HEK293 cells (Fig. 4C).

The Bmi-1 amino acid sequence contains at least two motifs, the RING finger and helix–turn–helix–turn–helix–turn (HTHTHT), and one KRMK nuclear-targeting sequence (NLS). The centrally located HTHTHT motif is necessary for transcriptional suppression. The RING finger motif and the nuclear localization signal correlate with the ability of Bmi-1 to induce transformation. To identify the region in Bmi-1 that associates with LSD1, we generated three deletion mutants of Bmi-1 [17]: deletion of the RING finger (Flag-Bmi-1 ΔRF), the helix–turn–helix motif (Flag-Bmi-1 ΔHT), or the KRMK NLS (Flag-Bmi-1 ΔNLS) (Fig. 4D). When these deletion mutants of Bmi-1 were coexpressed with LSD1 in HEK293 cells, we found that only ΔHT-Bmi-1 was able to interact with LSD1, indicating that the RF and NLS regions of Bmi-1 are responsible for its interaction with LSD1 (Fig. 4D).

The N-terminal third of LSD1 contains a SWIRM domain, and the C-terminal two-thirds of LSD1 comprise an amine oxidase (AO) domain that shares extensive sequence homology with FAD-dependent amine oxidase (Fig. 4E) [16]. To identify the region that is responsible for the LSD1 interaction with Bmi-1, we generated LSD1 domain-deletion mutants and coexpressed them with Bmi-1 in HEK293 cells. Immunoprecipitation of Bmi-1 revealed an association with full-length LSD1. A small C-terminal deletion mutant of LSD1 and the AO domain retained the ability to interact with Bmi-1 (Fig. 4E). The N-terminal region of the SWIRM domain, however, was incapable of interacting with Bmi-1. These results indicate that the AO domain is required for the interaction of LSD1 with Bmi-1. Together, these data indicate that Bmi-1 interacts with LSD1 and that this interaction is mediated through the AO domain of LSD1 and the RF and NLS regions of Bmi-1.

### LSD1 inhibition upregulates PDL1 expression and induces T cell suppression

**Fig. 5:**
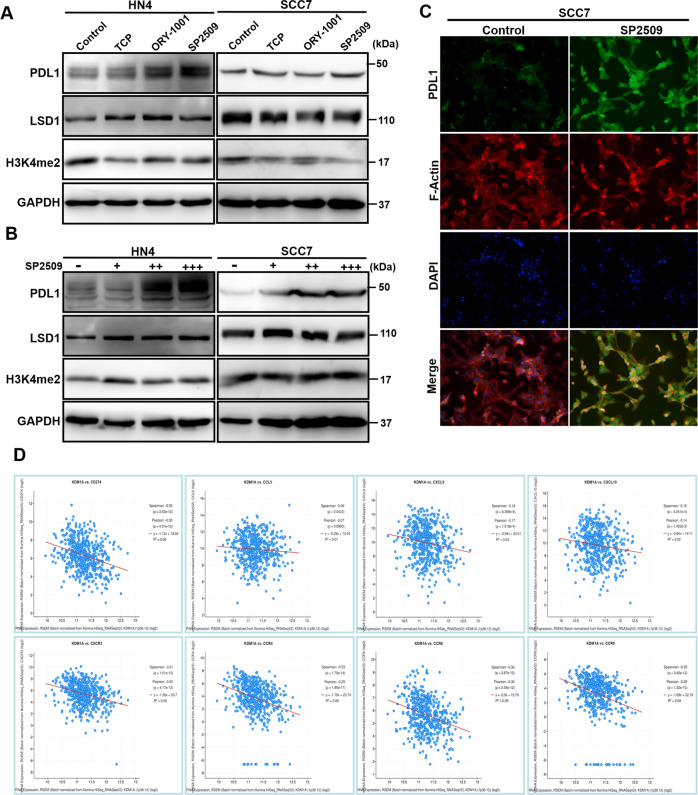
Targeting LSD1 induces PDL1 expression in HNSCC. **A** Western blot analysis of PDL1, LSD1, and H3K4me2 expression in HN4 and SCC7 cells treated with three different LSD1 inhibitors (50 µM TCP, 2.5 µM ORY-1001, 2.5 µM SP2509) for 24 h. **B** Western blot analysis of PDL1, LSD1, and H3K4me2 expression in HN4 and SCC7 cells treated with different concentrations of the LSD1 inhibitor SP2509 (0, 1.25, 2.5, 5 µM) for 24 h. **C** Images of immunofluorescence staining for PDL1 expression in SCC7 cells treated with or without SP2509 (1.25 µM) for 24 h; nuclei were stained with DAPI (blue). **D** Pearson correlation coefficients between immunoregulatory factors and LSD1 across HNSCC samples from the TCGA database.

To study the functional role of LSD1, most studies have used either in vitro cell culture systems or transplanted human cancer cells in immunodeficient mice but have not explored the role of LSD1 in regulating tumor responses to host immunity. To further investigate the tumor–host immunity interaction, we examined the tumorigenicity of mouse-derived SCC7 cells treated with LSD1 inhibitors in both immunodeficient nude mice and immunocompetent C3H mice. As expected, in nude mice, tumor volume and tumor weight were both significantly reduced in the group treated with an LSD1 inhibitor (SP2509) compared with the control group (Fig. S2a). However, in C3H mice, there was no significant difference in tumorigenicity between the group treated with the LSD1 inhibitor (SP2509) and the control group (Fig. S1a). PDL1 expressed on the cell surface of cancer cells exerts immunosuppressive effects by binding to the PD-1 receptor on activated T cells. Using flow cytometry, we found that PDL1 expression was increased in subcutaneously inoculated tumor tissue treated with the LSD1 inhibitor (Fig. S2b). We also observed that exhaustion of infiltrated CD8^+^ T cells, reflected by PD-1^+^ and TIM3^+^ FACS staining, was significantly increased (Fig. S2b). To further determine whether inhibition of LSD1 affects the PDL1 protein level, we first examined the effects of LSD1 inhibitors on growth of HN4 and SCC7 cells. Impressively, we found that both HN4 and SCC7 cells were much more sensitive to SP2509 than TCP and ORY1001 (Fig. S3). Then we treated HN4 cells and mouse SCC7 cells with the three LSD1 inhibitors and determined PDL1 expression by immunoblotting. The LSD1 inhibitors induced the expression of PDL1 in a dose-dependent manner in both HN4 and SCC7 cells (Fig. 5A, B). IF examination of PDL1 in SCC7 cells also revealed upregulated expression after SP2509 treatment (Fig. 5C). Moreover, the correlations between the expression of LSD1 (KDM1A) and immune signature genes such as CD8^+^ T cell-attracting chemokines (CCL5, CXCL9, CXCL10, CXCR3, CXCR4, CXCR6, and CXCR8) and the immune checkpoint molecule PDL1 (CD274) were evaluated in a cohort of HNSCC clinical specimens in The Cancer Genome Atlas. We observed negative correlations between LSD1 and immune-related genes (Fig. 5D). Therefore, our results suggest that LSD1 inhibition can upregulate PDL1 expression and induce immunosuppression in HNSCC.

### Inhibition of LSD1 enhances the efficacy of PD-1 blockade in vivo

**Fig. 6:**
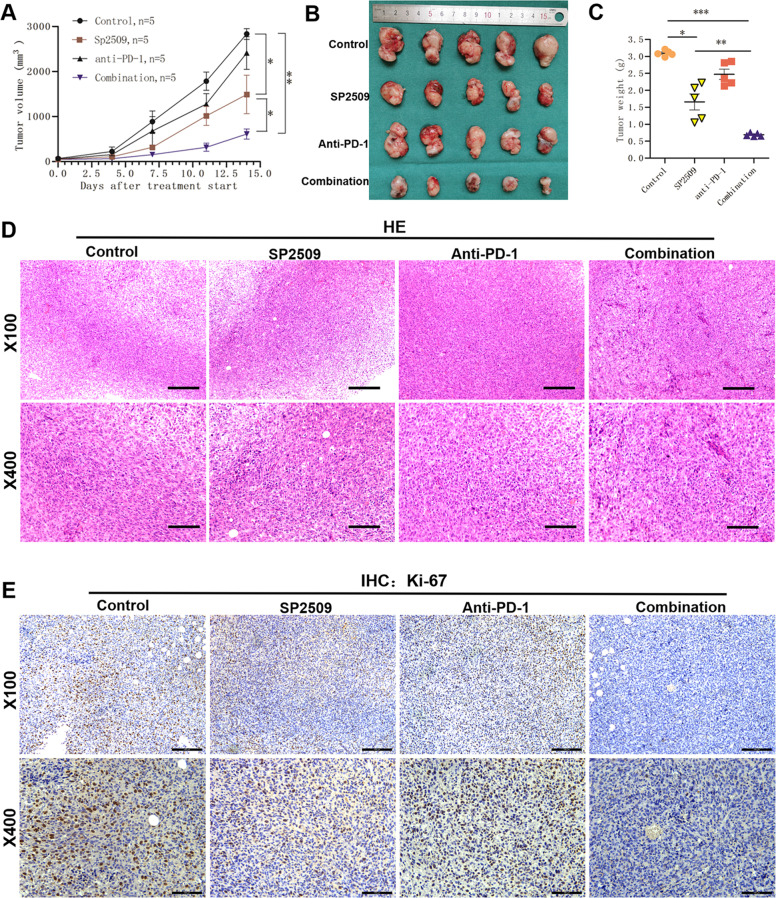
Inhibition of LSD1 enhances the efficacy of PD-1 blockade in vivo. **A** Mouse SCC7 cells were injected into C3H mice. When established tumors were palpable, the mice were treated with vehicle, SP2509, an anti-PD-1 mAb, or the combination of SP2509 and the mAb (*n* = 7) via i.p. injection. Tumors were measured with calipers, and values were plotted. The vertical bars indicate the mean tumor size (mm^3^) ±SE. **B** SCC7 tumors in each group were harvested and photographed at the end of the experiment. Photographs of the xenograft tumors are shown. **C** Tumor weights were measured for each treatment group at autopsy. **D** Representative HE staining of SCC7 xenograft tumors treated with vehicle, SP2509, the anti-PD-1 mAb, or SP2509 + anti-PD-1 mAb. **E** Representative immunohistochemical staining for Ki-67 in SCC7 xenograft tumors treated with vehicle, SP2509, the anti-PD-1 mAb, or SP2509 + anti-PD-1 mAb. (*p < 0.05, **p < 0.01, ***p < 0.001).

Our data demonstrated that LSD1 inhibition stabilizes PDL1 for immune evasion. Consequently, we sought to determine whether targeting LSD1 could potentate the antitumor efficacy of PD-1 blockade in vivo. We evaluated LSD1 inhibitor and anti-PD-1 treatments alone or in combination in the SCC7 syngeneic mouse model. The combined treatment demonstrated a better therapeutic benefit than either monotherapy (Fig. 6A–C). PD-1 blockade as a single-agent approach achieved no discernible difference in tumor progression versus control treatment. Moreover, there were significantly fewer Ki-67-positive tumor cells with the combined treatment than with either treatment alone when SCC7 tumors were evaluated (Figs. 6E and S4). Mice that received the combination treatment did not show any significant changes in body weight. These findings demonstrate the potential to improve treatment efficacy by cotargeting the LSD1 and PD-1/PDL1 axes in HNSCC patients.

### Anti-PD-1 therapy in combination with LSD1 blockade improves antitumor activity by overcoming PDL1-mediated immune escape

**Fig. 7:**
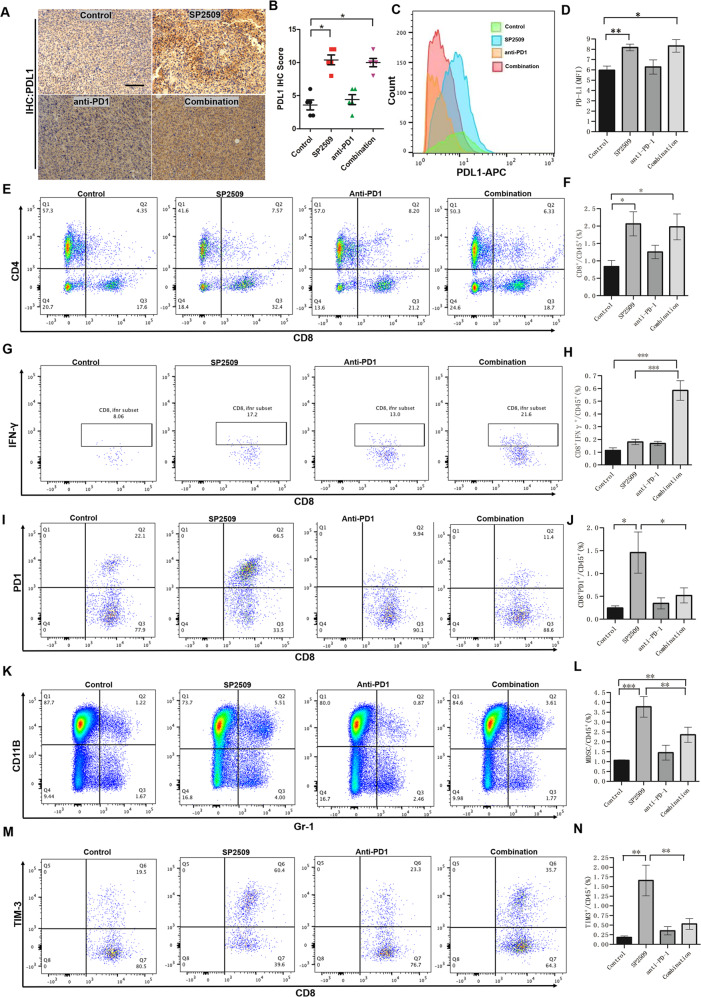
Characterization of the immune cell profile of tumor tissues after SP2509 inhibition and anti-PD-1 treatment. **A**–**D** Both flow cytometric analysis and IHC staining were used to identify the changes in PDL1 expression in tumor tissues after SP2509 inhibition and anti-PD-1 treatment. **A** Representative IHC images of PDL1 in tumor sections from each treatment group. **B** The histograms show that the percentages of PDL1-positive cells per field were similar between each treatment group and the untreated group. **C** Representative FACS images of PDL1 in tumor cells from each treatment group. **D** The histograms show the percentages of PDL1-positive cells in each treatment group based on FACS analysis. Both SP2509 treatment alone and combination treatment with SP2509 and the anti-PD-1 antibody resulted in a significant increase in PDL1 expression in tumor tissue compared with vehicle treatment. **E**–**N** Numbers of different subsets of immune cells in tumor tissue determined from the percentage of the total immune cell population (CD45^+^) of mice treated with the inhibitor SP2509 and PD-1 blockade versus vehicle based on FACS analysis. **E**, **F** Number of CD4^+^ T cells. **G**, **H** Number of CD8^+^IFNγ^+^ T cells. **I**, **J** Number of CD8^+^PD-1^+^ T cells. **K**, **L** Number of MDSCs. **M**, **N** Number of CD8^+^TIM-3^+^ T cells. n.s., not significant; *P < 0.05; **P < 0.01; ***P < 0.001.

We next characterized the effect of treatment on immune cell populations in the TME by flow cytometry and immunochemical staining. Consistent with the in vitro results, we showed that SP2509 treatment alone resulted in a significant increase in PDL1 expression in subcutaneously inoculated tumor tissue compared with vehicle treatment (Fig. 7A, B). Moreover, we also isolated SCC7 cells from transplanted tumors and performed flow cytometry to detect cell-surface PDL1 expression, which was higher in the SP2509 treatment group (Fig. 7C, D). HNSCC tumors are highly immunosuppressive and characterized by impaired T cell function and immunosuppressive cell accumulation. Thus, we next examined the effect of SP2509 treatment on antitumor immunity. Compared with the vehicle treatment groups, the SP2509 monotherapy and combined treatment groups showed markedly enhanced infiltration of CD8+ T cells into tumors (Figs. 7E, F and S5b, c). However, the proportions of immunosuppressive CD8^+^PD-1^+^ T cells, CD8^+^TIM3^+^ T cells, and myeloid-derived suppressor cells were increased in the SP2509 single-agent treatment group but decreased in the combination group (Fig. 7G, N). Moreover, we found that the proportion of cytotoxic CD8^+^IFNγ^+^ T cells was significantly increased in the combination treatment group compared with all other treatment groups. TUNEL assay showed that apoptotic cells were significantly increased in tumor section from the combined group (Fig. S5d, e). All these results indicated that the combination therapy enhanced antitumor immunity in mice. To our surprise, we observed that the combined treatment increased the proportion of regulatory T cells in the TME, whereas there were no significant changes in other cell populations (Fig. S5a). Thus, these data reveal that inhibition of LSD1 may improve antitumor immunity by increasing effector T cells in HNSCC.

## Discussion

**Fig. 8:**
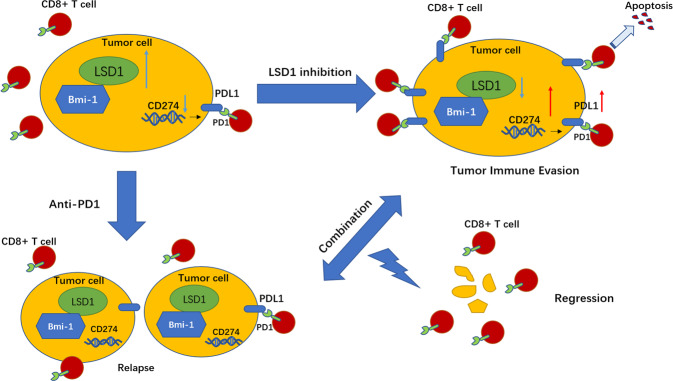
A proposed model showing a novel therapeutic strategy for HNSCC patients by using a combination of LSD1 inhibition and PD-1 blockade. High expression of LSD1 promotes the CSC properties by regulating Bmi-1 expression. Inhibition of LSD1 induces the upregulation of PDL1 levels and promotes tumor immune evasion. Combination of LSD1 inhibitor and anti-PD-1 monoclonal antibody can overcome tumor immune evasion and greatly inhibit tumor growth, which providing a novel and promising combined strategy for the treatment of HNSCC.

In this study, we provided a model (Fig. 8) showing that LSD1 was overexpressed in HNSCC cancer cells and conferred CSC-like features mediated through Bmi-1 expression to them. Targeting LSD1 suppressed CSC characteristics and inhibited tumorigenicity in immunodeficient xenografts. However, this suppression induced the upregulation of PDL1 levels, which compromised antitumor immunity and reduced antitumor efficacy in an immunocompetent mouse model. Functionally, the combination of an LSD1 inhibitor and an anti-PD-1 mAb could overcome tumor immune evasion and greatly inhibit tumor growth, which was associated with reduced Ki-67 levels and augmented CD8^+^ T cell infiltration in immunocompetent tumor-bearing mouse models. In summary, these findings provide a novel and promising combined treatment strategy for HNSCC using a combination of LSD1 inhibition and PD-1 blockade.

HNSCC is a heterogeneous tumor with high metastasis and recurrence rates, and there has been no significant improvement in 5-year survival over the past decades [25]. Several studies have revealed that LSD1 is an essential oncogene driving cancer initiation, overgrowth, and metastatic dissemination in multiple contexts, including HNSCC [12, 26]. Our results provide clues that LSD1 is essential for the maintenance of CSC-like characteristics in HNSCC. Moreover, we demonstrated that Bmi-1 is required for LSD1-driven HNSCC oncogenesis. Bmi-1 is an important stem cell self-renewal factor that has been found to be abnormally expressed in HNSCC and might be associated with the self-renewal of CSCs in HNSCC [27]. A previous study reported that endothelial cell-derived growth factors potently promote the survival and self-renewal of CSCs in HNSCC by upregulating Bmi-1 [28]. Cisplatin treatment has been found to induce Bmi-1 expression and increase CSC populations in HNSCC [29]. In human HNSCC, Twist1 and Bmi-1 act cooperatively to induce EMT and stemness, thereby indicating a role for Bmi-1 in HNSCC metastasis [30]. Moreover, abnormal activation of AP-1 plays a critical role in Bmi-1^+^ CSC-mediated HNSCC invasive growth, and targeting the tumor bulk and Bmi-1^+^ CSCs by combination therapy effectively inhibits HNSCC growth and eliminates metastases [31]. In our study, we found that LSD1 is essential for the maintenance of CSC-like characteristics through Bmi-1 in HNSCC. Moreover, our results indicate that Bmi-1 interacts with LSD1 and that this interaction is mediated through the AO domain of LSD1 and the RF and NLS regions of Bmi-1. Thus, our study extends these findings, confirms the importance of the LSD1-Bmi-1 complex in HNSCC, and reinforces the view that Bmi-1 is a key characteristic of HNSCC CSCs.

Immune checkpoint blockade via an anti-PD-1 or anti-PDL1 mAb has produced substantial benefits in several advanced cancers. However, the overall response rates of anti-PD-1 or anti-PDL1 mAb therapy rarely exceed 40%. Therefore, the development of alternative therapeutic strategies, including combination therapies, has been heavily investigated [32]. Accumulating evidence indicates that abnormal epigenetic modifications play important roles in silencing the expression of effector T cell chemokines in cancer [33]. LSD1 depletion enhances tumor immunogenicity and T cell infiltration in poorly immunogenic tumors and elicits significant responses to anti-PD-1 therapy in checkpoint blockade-refractory mouse melanoma [14]. In triple-negative breast cancer, LSD1 plays an important role in mediating epigenetic reprogramming that alters the T cell landscape. The combined use of an LSD1 inhibitor effectively enhances the therapeutic efficacy of anti-PD-1 immunotherapy [22]. In this study, we treated HNSCC cells with different LSD1 inhibitors and assessed PDL1 expression by immunoblotting. The LSD1 inhibitors induced the expression of PDL1 in HNSCC cells in a dose-dependent manner. Moreover, we evaluated an LSD1 inhibitor and anti-PD-1 treatment combination in the SCC7 syngeneic mouse model. The combined treatment demonstrated better therapeutic benefits than either treatment alone. In conclusion, we demonstrated that LSD1 conferred HNSCC with CSC-like features through Bmi-1 expression. Inhibition of LSD1 induced PDL1 expression and tumor immune evasion. Moreover, our studies identify a new strategy for targeting crosstalk between epigenetic modulators and immune compartments as a novel therapeutic strategy for HNSCC patients with a poor immune response.

## Supplementary information

Supply Figure 1-5

Supply Figure legends

## Data Availability

The datasets used and/or analyzed during the current study are available from the corresponding author on reasonable request.
