# Supplementary figures and images for: Targeting LSD1 suppresses stem cell-like properties and sensitizes head and neck squamous cell carcinoma to PD-1 blockade
Source: Cell Death Dis. 2021 Oct 23;12(11):993. doi: 10.1038/s41419-021-04297-0 (PMC8542042; doi:10.1038/s41419-021-04297-0)

# Supply Figure 1

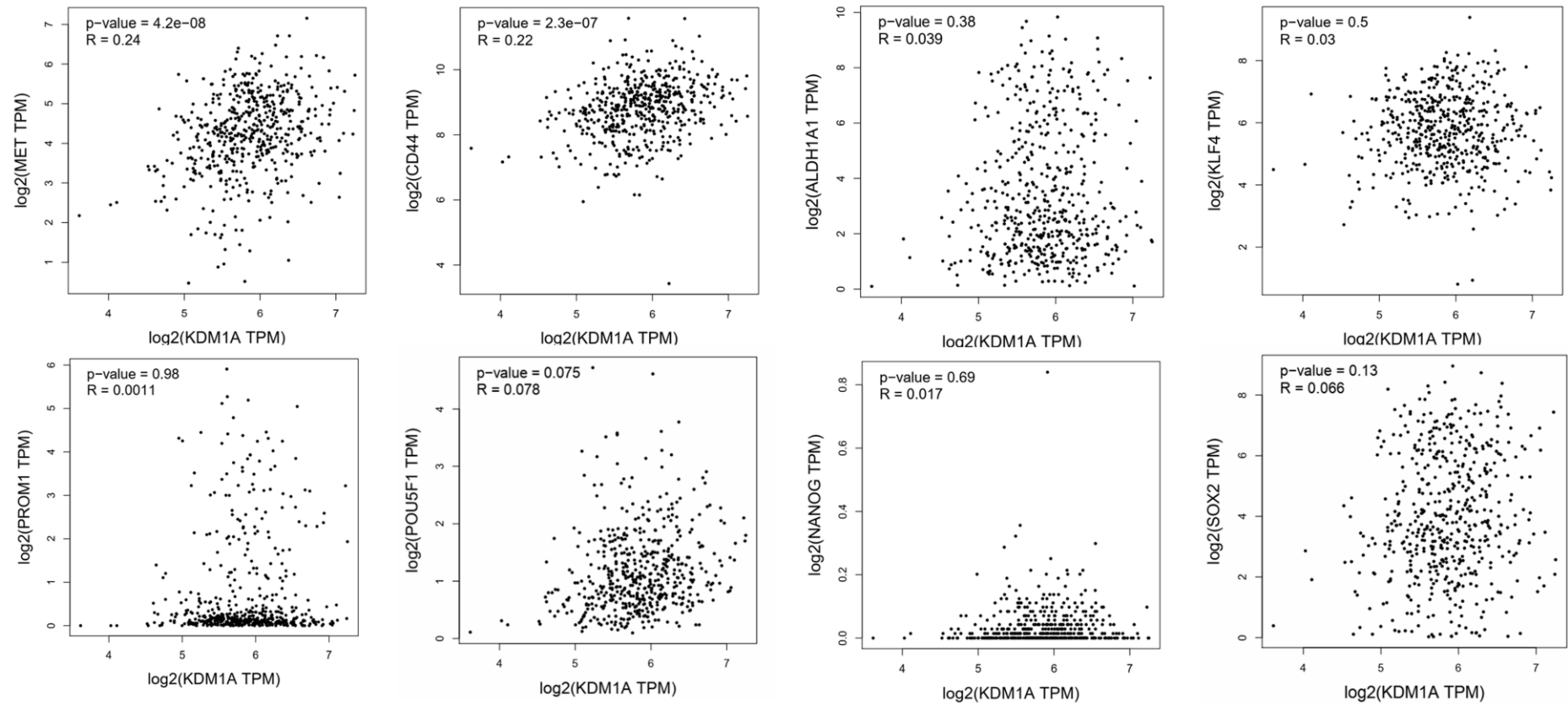

Supply Figure 2

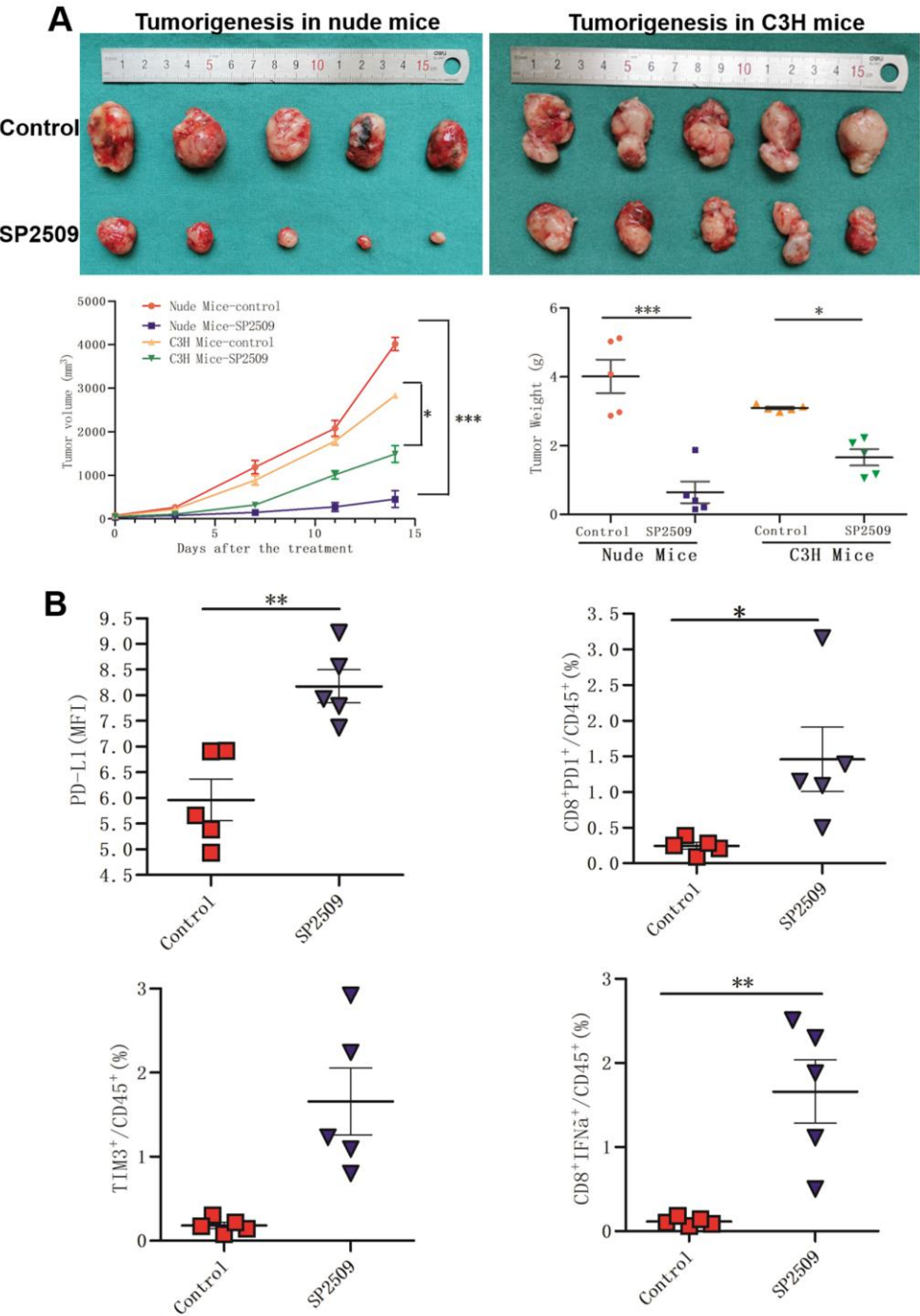

Supply Figure 3

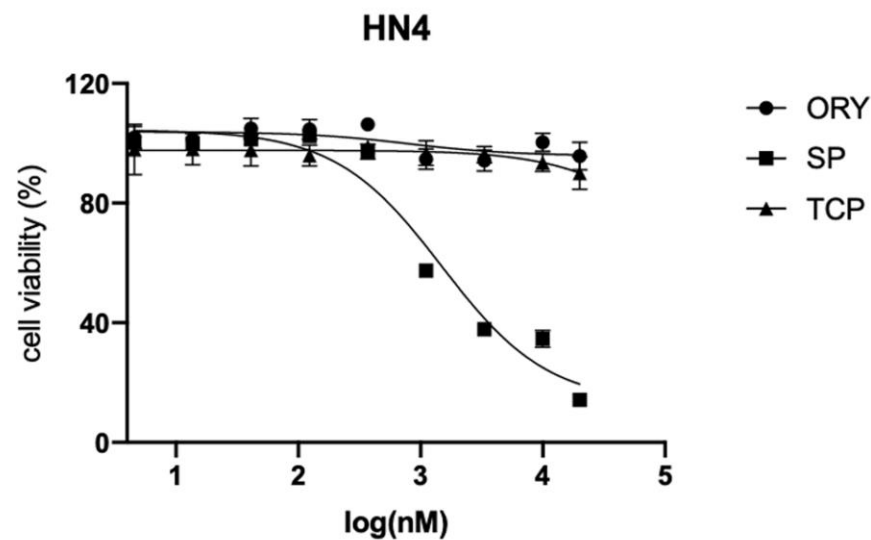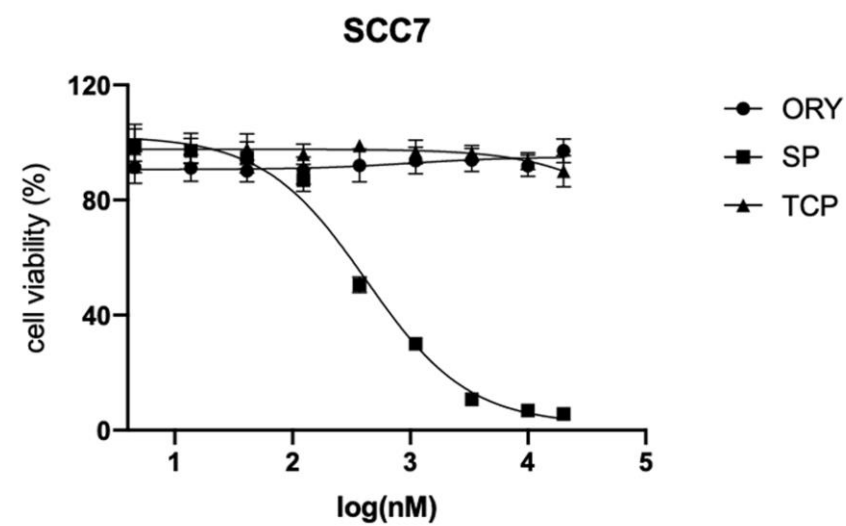

Supply Figure 4

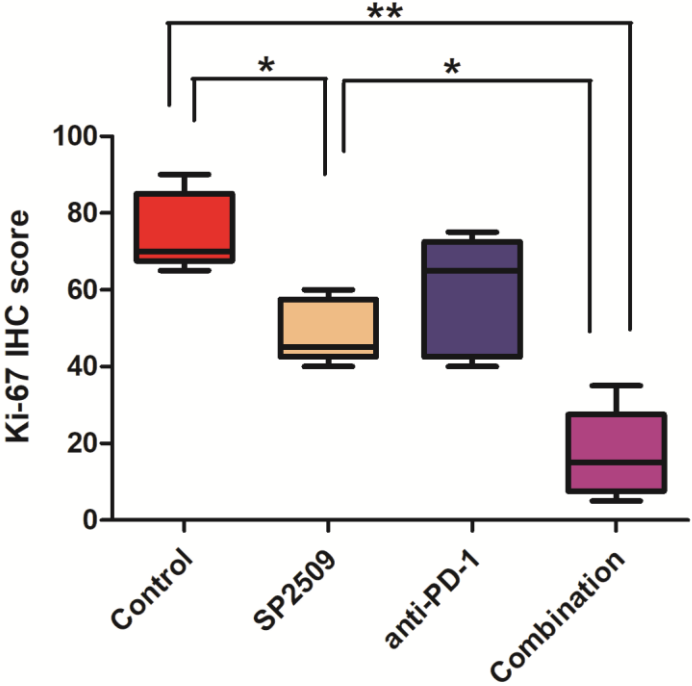

Supply Figure 5

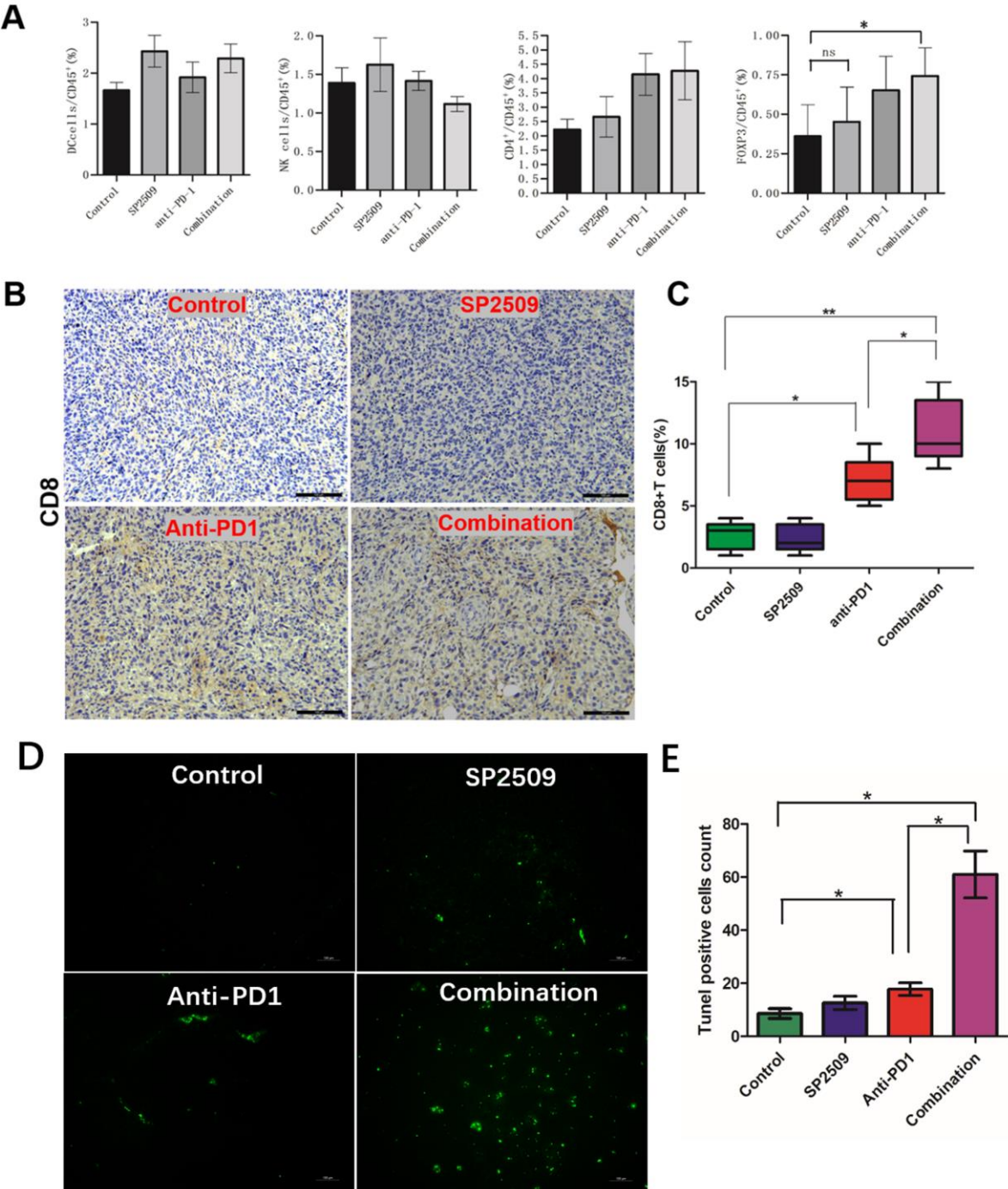

Supplement: Supplementary file 1 — Supply Figure 1-5 [file 41419_2021_4297_MOESM1_ESM.pdf]
