# Supplementary material for: Targeting LSD1 suppresses stem cell-like properties and sensitizes head and neck squamous cell carcinoma to PD-1 blockade
Source: Cell Death Dis. 2021 Oct 23;12(11):993. doi: 10.1038/s41419-021-04297-0 (PMC8542042; doi:10.1038/s41419-021-04297-0)
Supplement: Supplementary file 2 — Supply Figure legends [file 41419_2021_4297_MOESM2_ESM.docx]

**Supply Figure legends**

**Figure S1. (related to Figure 3)**

Correlations between the expression levels of LSD1 and different CSC-related genes in HNSCC.

**Figure S2.** **LSD1 inhibition upregulates PDL1 expression and induces T cell suppression. (related to Figure 5)**

**a** Tumor images (upper), tumor growth (lower, left) and tumor weight (lower, right) of immunocompetent or immunodeficient mice after SP2509 inhibition. The differences in tumor volume or weight between the SP2509 treatment group and the control group in the nude mouse experiment were significantly higher than those in the C3H mouse experiment. **b** LSD1 inhibition induced upregulation of PDL1 expression and increased the proportions of CD8+PD-1^+^T cells, CD8^+^TIM-3^+^ T cells and CD8^+^IFNγ^+^ T cells. * p < 0.05, ** p < 0.01, *** p < 0.001.

**Figure S3. (related to Figure 5)**

HN4 and SCC7 (96-well plate) were treated with TCP, ORY or SP2509 as indicated. Cellular viability was assayed using the CCK8 kit.

**Figure S4. (related to Figure 6)**

IHC scores for Ki-67 expression in tumor sections from each treatment group.

**Figure S5. Characterization of the immune cell profile in tumor tissues after SP2509 inhibition and anti-PD-1 treatment. (related to Figure 7)**

**a** Combined treatment increased the proportion of Tregs in the TME, whereas there were no significant changes in DC or NK cell populations among different treatment groups.

**b, c** IHC staining was used to identify the changes in CD8^+^ T cells in tumor tissues after SP2509 inhibition and anti-PD-1 treatment. Combined treatment significantly increased the infiltration of CD8^+^ T cells into the TME compared with the other treatments. * p < 0.05, ** p < 0.01.

**d** Representative photographs of TUNEL staining in different groups. Scale bars=100 μm.

**e** Quantitative analysis of TUNEL-positive cells content among groups. (n=5, *p＜0.05)
